# P016 Calcineurin inhibitor-induced pain syndrome: a rare presentation of debilitating lower limb pain

**DOI:** 10.1093/rap/rkag113.046

**Published:** 2026-11-03

**Authors:** Matt Taylor, Chiara Giacomassi

**Affiliations:** Imperial College Healthcare NHS Trust, London, United Kingdom; Imperial College Healthcare NHS Trust, London, United Kingdom

## Abstract

**Introduction:**

This case describes a rare presentation of calcineurin inhibitor-induced pain syndrome in a patient with a recent liver transplant who was referred to rheumatology for vertebral fractures. The diagnosis was made from the combination of bilateral symmetrical severe lower limb pain, allodynia and hyperalgesia around three months after starting tacrolimus, alongside supportive MRI findings of patchy bone marrow (BM) oedema, and a significant improvement in symptoms on switching to an alternative calcineurin inhibitor.

**Case description:**

This 52 year old patient was referred to the rheumatology clinic regarding osteoporosis management. She underwent a DBD liver transplant for alcohol-related liver disease and was started on immunosuppressive treatment including prednisolone, tacrolimus and mycophenolate mofetil. She developed acute atraumatic pain in the spine and left ribcage two months later whilst on high dose steroids. She had sustained multiple vertebral fractures (T5, T7, T10, T11, T12, L2).

During her clinic appointment it was noted that she had developed marked allodynia in both lower limbs starting around 10-12 weeks after her transplant, gradual onset, worse at night with sleep disturbance. There was severe allodynia and hyperalgesia on palpation of ankles, shins and knees, with mild swelling of the left knee and both ankles. Lower limb neurological and vascular examination was normal. There was no upper limb involvement.

Inflammatory markers, calcium, phosphate, vitamin D, serum protein electrophoresis, rheumatoid factor, anti-CCP antibody and ANA were all within normal limits. Alkaline phosphatase was elevated at 166 units/L (although improving post-transplant). Parathyroid hormone was elevated at 17pmol/L. During the peak of her symptoms in February, the tacrolimus level was 15.9ng/mL.

MRI of both knees and ankles noted non-specific focal BM oedema at the left fibular head and patchy BM oedema bilaterally within the calcaneum and cuboid. There was no evidence of avascular necrosis or inflammatory arthritis. MRI of the thoracolumbar spine did not show any cord change or nerve compression to explain the patient’s symptoms.

This presentation with symmetrical lower limb allodynia post-transplant with BM oedema on imaging was suspicious for calcineurin inhibitor-induced pain syndrome (CIPS). Tacrolimus was switched to ciclosporin with a notable improvement in her bilateral lower limb pain. She was given IV zoledronic acid for management of osteoporosis, and also to curb the underlying osteoclast activity and to provide analgesia.

**Discussion:**

CIPS is a rare and poorly understood complication of calcineurin inhibitor therapy. This case outlines several typical features of this syndrome, with severe lower limb pain (particularly in the feet, ankles and knees) developing around 3 months after starting calcineurin inhibitors, with allodynia and hyperalgesia also previously reported.

Diagnosis of CIPS requires good awareness of the condition to make the connection between the initiation of calcineurin inhibitor therapy and the delayed onset of otherwise unexplained severe lower limb pain. It is vital to exclude other important differentials, including neurological and vascular disorders that might present similarly.

There are a number of possible mechanisms described, considering the effects of calcineurin inhibitors on pain modulation, vascular permeability and bone turnover.

Blood tests are often normal. The elevated alkaline phosphatase can be a helpful, although of difficult interpretation in patients with known liver disease. Bone-specific alkaline phosphatase could be used to help differentiate from a liver or gastrointestinal pathology, although was not felt to be necessary in this case.

The presence of patchy (BM) oedema (seen here at the left fibular head and bilaterally within the calcaneum and cuboid) on MRI was a helpful finding in this case and helped to support the clinical diagnosis. MRI was also supportive in assessing for differential diagnoses in this case, including inflammatory arthritis and osteonecrosis. Furthermore, spinal imaging was also important in this case in the context of the known vertebral fractures to look for any cord compromise or radiculopathy.

Symptoms often respond to reductions in dose or switching to alternative calcineurin inhibitor therapy. The rapid improvement in symptoms on switching the calcineurin inhibitor is typical of the condition, although not well understood. Other treatments aimed at targeting pain modulation, vasoconstriction, and high bone turnover have been put forward as possible therapeutic options.

**Key learning points:**

• To diagnose CIPS requires a good awareness of the condition so that you can make the link between the initiation of calcineurin inhibitor therapy and the delayed onset of severe lower limb pain.

• It is vital to rule out other conditions (musculoskeletal, vascular and neurological). MRI in particular is a sensitive and useful tool in making the correct diagnosis.

• It is interesting to observe how significantly symptoms improve with reductions in dose of the calcineurin inhibitor, or switching to an alternative calcineurin inhibitor as we did in this case. Switching immunosuppressive agents in patients with a recent liver transplant requires careful consideration and strong multidisciplinary team working.

• There are several proposed mechanisms for the development of CIPS that represent multiple avenues for further study. In particular in our case, the reported effects on vascular permeability and osteoblast activity are interesting in the context of the atraumatic vertebral fractures our patient sustained.

• Rheumatology are well-placed in managing metabolic bone disorders and pain syndromes in the context of patients with complex multisystem disease, and this case helped me to reflect on the value that rheumatology can offer in diagnosing and managing patients with CIPS in future.

• One of the key learning points for me to gain from the conference is to gain a better understanding for how rare metabolic bone disorders present and are identified, and about the interactions between rheumatology and other specialities in the diagnosis and management of these patients with complex and rare disease.

